# Perforated acute appendicitis with no peritonitis in a premature baby: a case report

**DOI:** 10.1186/s13256-017-1289-0

**Published:** 2017-05-05

**Authors:** Fayza Haider, Barrak Ayoub, Mariam Al Kooheji, Mona Al Juffairi, Safa Al-Shaikh

**Affiliations:** 1 Department of Surgery-Pediatric Surgery Unit, Salmaniya Medical Complex, P.O. Box 12, Manama, Bahrain; 2 Department of Pediatrics, Salmaniya Medical Complex, Manama, Bahrain; 3 Department of Pathology, Salmaniya Medical Complex, Manama, Bahrain

**Keywords:** Case report, Neonatal appendicitis, Peritonitis, Necrotizing enterocolitis, Premature baby, Preterm, Pneumoperitoneum

## Abstract

**Background:**

Acute appendicitis in a neonate and premature baby is still considered a rare entity as diagnosis is always made after surgical exploration for acute abdominal findings mimicking necrotizing enterocolitis.

Our reported case is a premature baby who had a perforated appendix with no evidence of peritonitis.

**Case presentation:**

We describe the case of a premature Bahraini girl born at 29 weeks of gestation by spontaneous vaginal delivery to a 39-year-old G6P5 mother. She was kept on a ventilator for the first 6 days of life, and had an uneventful Neonatal Intensive care stay until her 47th day of life when she developed sepsis that required ventilator support for 3 days. At day 51 she developed abdominal distension and was referred to a pediatric surgeon by day 54 with pneumoperitoneum. Her abdomen was soft with minimal tenderness and no evidence of erythema or edema. In view of pneumoperitoneum and previously reported sepsis, she was taken for exploratory laparotomy. The findings were consistent with a perforated appendix with no evidence of peritonitis or necrotizing enterocolitis. An appendectomy was performed. She had a smooth postoperative recovery.

**Conclusions:**

Neonatal appendicitis continues to be a diagnostic challenge. Only with a high index of clinical suspicion and teamwork can these cases be managed successfully and mortality and morbidity rates may reduce.

## Background

Although no age is free from the risk of appendicitis, it is extremely uncommon in newborns [1].

The rarity of neonatal appendicitis (NA) together with lack of specific signs and low index of suspicion has led to delay in diagnosis and surgical intervention [2]. Most of the time the diagnosis is delayed and is made after perforation has occurred.

It was proposed that NA is actually a limited form of necrotizing enterocolitis (NEC) [3]. The observation that more than 50% of babies with appendicitis are preterm [4] adds strength to the theory because 90% of NEC is also found in premature babies [1].

Although it has been reported for over 100 years, the total collective cases reported are around 100 of which most presented with peritonitis [4].

## Case presentation

**Fig. 1:**
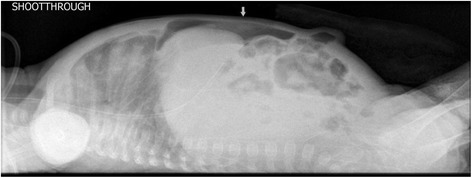
A shoot-through lateral abdominal film showing pneumoperitoneum (*arrow*)

**Fig. 2:**
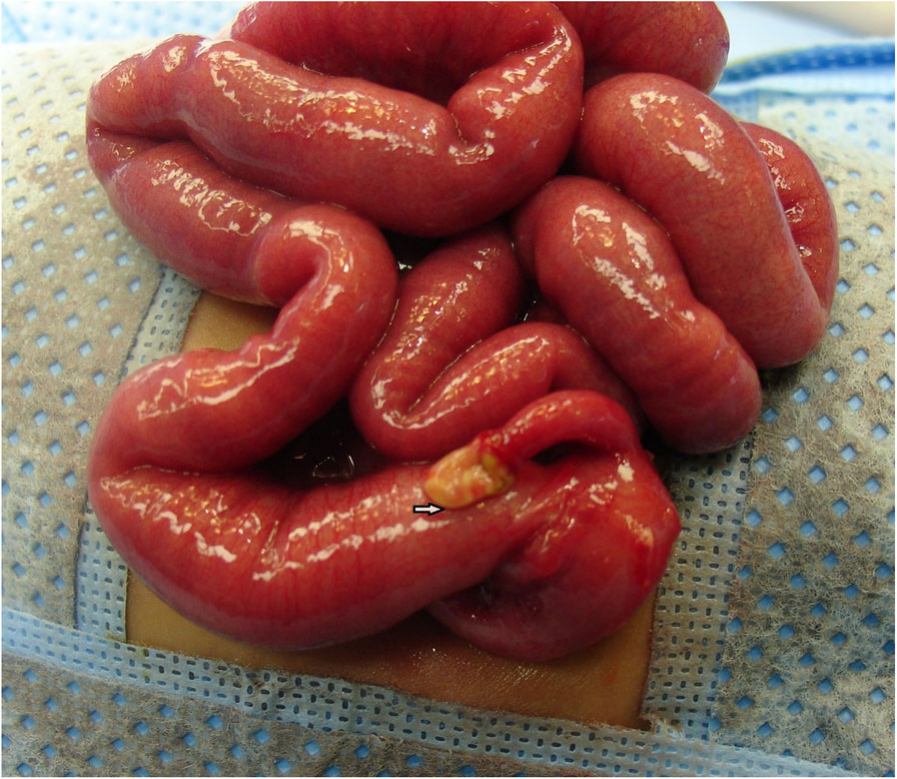
All the small bowel is healthy in appearance with perforated appendicular tip (*arrow*)

**Fig. 3:**
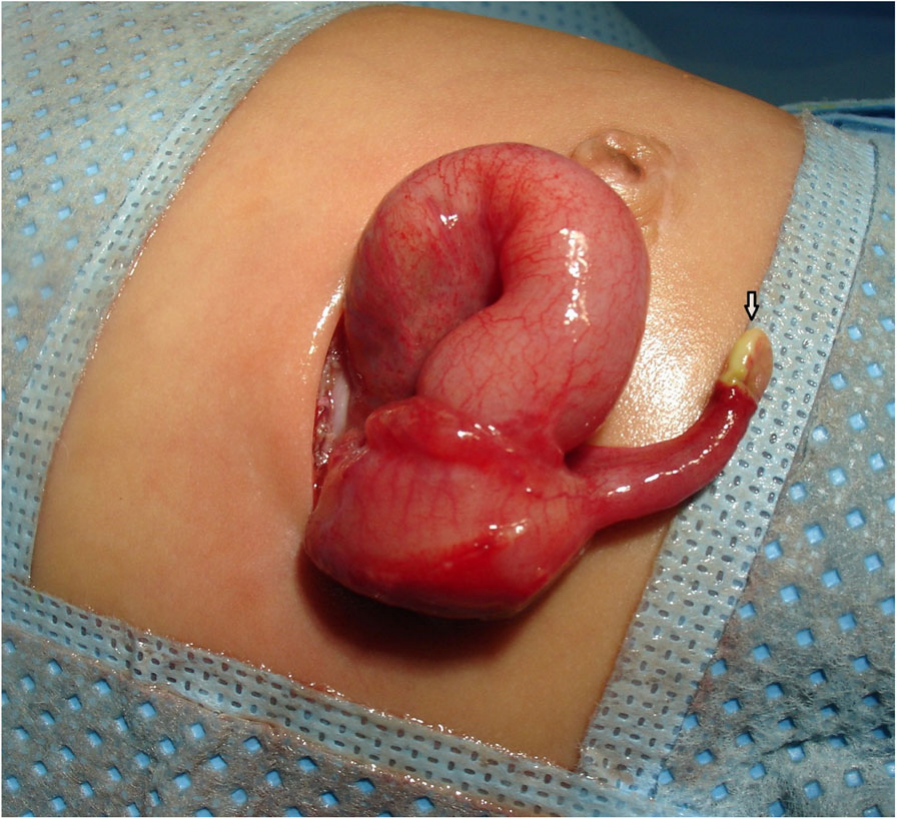
Appendicular tip perforation with no other evidence of gangrene (*arrow*)

**Fig. 4:**
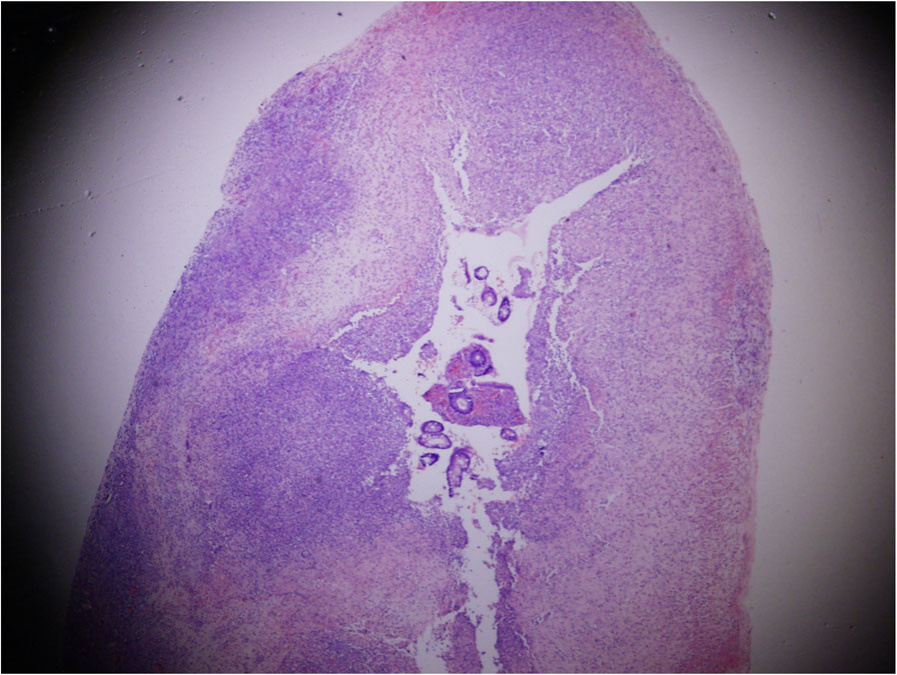
High-power view (hematoxylin and eosin stain) showing appendiceal wall with mucosal ulceration, marked acute transmural inflammation, and necrotic wall

**Fig. 5:**
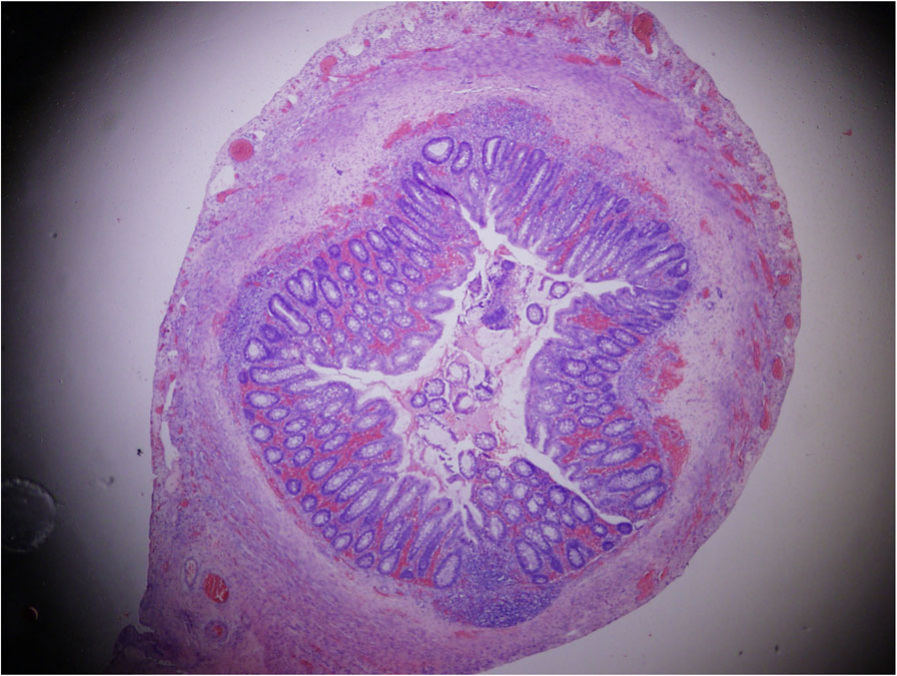
Low-power view (hematoxylin and eosin stain) showing congested appendiceal wall with intact mucosal lining

**Fig. 6:**
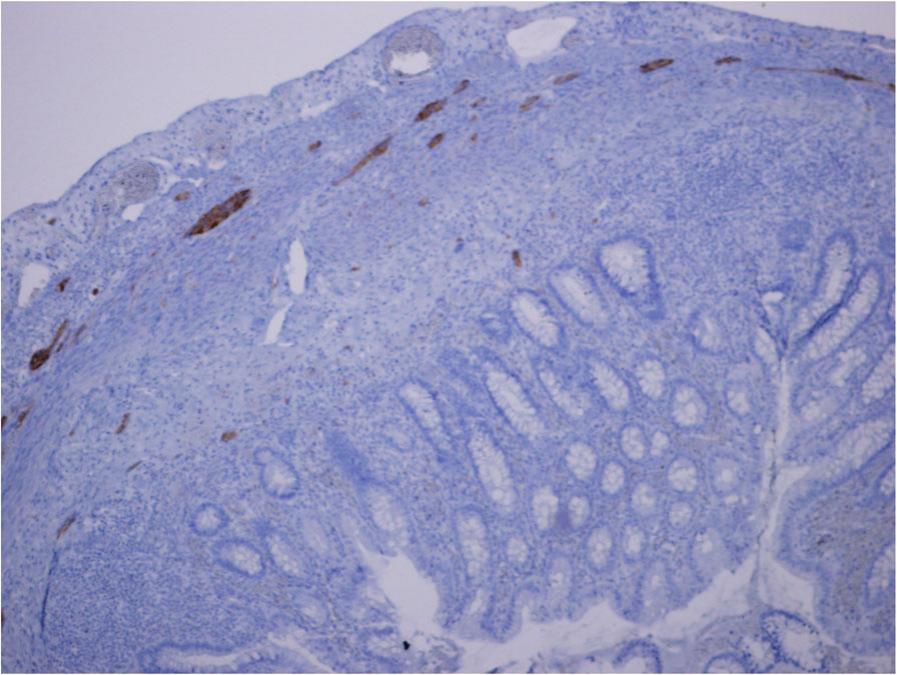
Neuron-specific enolase immunohistochemical stain showing positive staining for nerve fibers

We describe the case of a premature Bahraini girl born at 29 weeks of gestation by spontaneous vaginal delivery, to a 39-year-old gravida 6 para 5 mother. She was born “flat” with an APGAR score of 4 and 9 at 1 and 5 minutes respectively and a birth weight of 910 g. Ventilatory support was required during her first 6 days of life, with a dose of surfactant. She had an uneventful Neonatal Intensive care stay, tolerating breast milk and was gaining weight at a steady pace. On her 47th day of life she developed sepsis and required repeated ventilator support for 3 days. At day 51 she weighed 1400 g, but developed abdominal distension and feed intolerance. She was referred to the pediatric surgeon on day 54 when pneumoperitoneum was detected by a shoot-through lateral plain abdominal film (Fig. 1). Our examination showed her abdomen to be distended but soft with mild diffuse tenderness and no signs of peritonitis. There was no abdominal wall erythema or visible bowel loops. We did not find any hernias or abdominal masses. Her C-reactive protein (CRP) was 37 mg/L which is 12 times higher than the normal range. In view of pneumoperitoneum, she was taken for exploratory laparotomy. A classic right transverse upper abdominal incision was used to open her abdomen as the diagnosis was NEC. Her entire small and large bowel was healthy and normal in appearance (Fig. 2). Her appendix measured approximately 2 cm in length and was acutely inflamed with a perforation at the tip. Except for the site of perforation, there was no other evidence of gangrene (Fig. 3). The findings were consistent with a perforated appendix with no evidence of peritonitis or NEC. An appendectomy was performed. She had a smooth postoperative recovery where she was started on breast milk on the second postoperative day and her CRP was normal on the eighth postoperative day. Histology demonstrated mucosal ulceration of her appendiceal wall, marked acute transmural inflammation, and necrotic wall at the perforated end (Fig. 4) but the rest had intact mucosal lining (Fig. 5). A neuron-specific enolase (NSE) immunohistochemical stain showed positive staining for nerve fibers (Fig. 6) which excluded Hirschsprung’s disease. She was discharged home at day 120 of life and was thriving at 2-year follow-up.

## Discussion

NA is an extremely rare condition, with fewer than 50 cases reported in the last 30 years and just more than 100 over the last century [5]. The incidence of NA has been reported as 0.04 to 0.2% [6]. NA occurs in males approximately 75% of the time and 25 to 50% of all reported cases involve premature babies [1, 4]. As the survival of young babies improves with better perinatal care, one would expect the frequency of appendicitis to rise along with the incidence of NEC in this population [3].

The rarity of appendicitis in the neonatal period (0.04% reported incidence) is classically attributed to the broad orifice of the appendix (“conical,” “funnel,” or “fetal” anatomy), liquid diet, near-constant supine positioning, the lack of fecaliths in neonates, and the presumed relative infrequency of lymphatic hyperplasia in the periappendiceal region caused by lack of infectious stimuli [7]. The infrequency of this disease and resultant delay in diagnosis are largely responsible for the higher reported associated morbidity and mortality in perinatal and other age groups [4].

Generalized peritonitis and intestinal congestion of perforated appendicitis is difficult to distinguish from that of NEC [1]. Existing reports suggest that even when a misdiagnosis occurs, if that diagnosis nevertheless leads to surgical exploration, then the outcome is likely to be better than if laparotomy is delayed [7].

The most common presenting clinical feature in the literature was abdominal distension, which was present in 89% of patients [1] and it was the presenting feature in our patient. The presence of free air on plain abdominal radiograph may confer a more favorable prognosis by leading to early surgical intervention [5].

Pneumoperitoneum is the single most useful sign which was seen in 23 of the 44 (52%) patients with perforation. Even in the absence of correct clinical diagnosis, perforation – as indicated by pneumoperitoneum – appears to have prompted surgical exploration; this was the reason for the referral of our patient. NA does not appear to have any causal relationship with Hirschsprung’s disease [1] and our reported case has normal ganglia. The association between NA and NEC deserves further investigation because both share the same spectrum of risk factors [1].

Although rare, NA can be expected to occur with increasing frequency as perinatal conditions associated with appendicitis, such as prematurity and survival after severe hypoxia or ischemia, are much more common in this era of improved neonatal care [7].

## Conclusions

NA continues to be a diagnostic challenge. Only with a high index of clinical suspicion and teamwork can these cases be managed successfully and the mortality rate may reduce.
